# Bilateral subdural hygromas after endovascular coiling for ruptured aneurysmal subarachnoid hemorrhage: an unusual and rare complication

**DOI:** 10.1093/omcr/omab062

**Published:** 2021-08-13

**Authors:** Walid O Ahmed, Shady N Mashhour, Marwa E Abdelfattah

**Affiliations:** 1 Faculty of Medicine, Department of Critical Care Medicine, Cairo University, Cairo, Egypt; 2 Faculty of Medicine, Department of Radiology, Cairo University, Cairo, Egypt

## Abstract

Subarachnoid hemorrhage (SAH) with subdural hygroma (SH) was rarely reported after endovascular coiling. A 60-year-old male presented with impaired consciousness and convulsions due to SAH from a ruptured aneurysm. It was managed by endovascular coiling 20 h after the onset of symptoms. Serial brain imaging for 2 weeks revealed progressive bilateral SHs, more on contralateral side of leaking aneurysm. Management of SH was discussed in a multidisciplinary setting to be conservative as there was neither significant mass effect nor hydrocephalus. The patient recovered neurologically except for mild dysarthria. The SH persisted for 2 months and then cleared gradually. We concluded that SH may arise and become symptomatic as an unusual sequela of post-coiling of a ruptured intracranial aneurysm, in which the SH can complicate the clinical course of SAH. However, the symptomatic SH may resolve spontaneously and completely without any intervention, but needs meticulous neurological assessment and follow-up.

## INTRODUCTION

Collection of cerebrospinal fluid (CSF) in the subdural space can be described as ‘subdural hygroma’ (SH), ‘external hydrocephalus’ and ‘subdural effusion’. SH represents a unique clinical entity [1].

**Figure 1:**
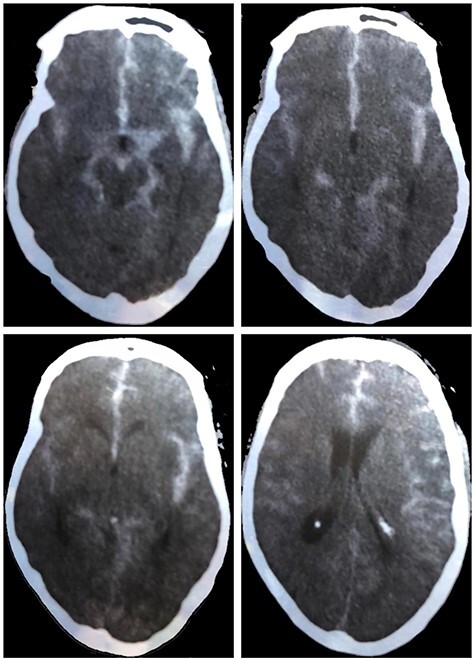
Unenhanced CT scan at the time of presentation demonstrating diffuse SAH with the main brunt of hemorrhage in the left suprasellar cistern and Sylvian fissure.

**Figure 2:**
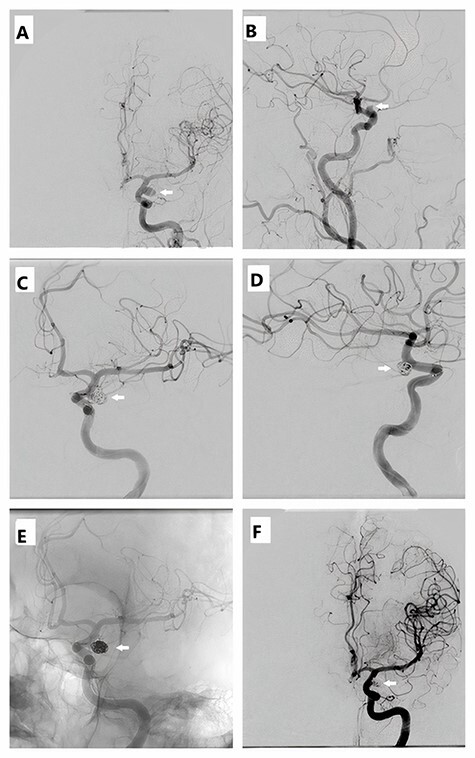
Cerebral angiography during endovascular coiling revealed: (**A**) a 4.5 × 6 mm left posterior communicating aneurysm with neck 2–3 mm (the source of current bleeding). (**B**) Another accidentally discovered 2.5 × 3.5 mm right para-ophthalmic aneurysm with a wide neck. (**C** and **D**) Left posterior communicating aneurysm (PCA) during coiling with the deployment of four coils. (**E** and **F**) Left PCA after final result and control angiograms.

**Figure 3:**
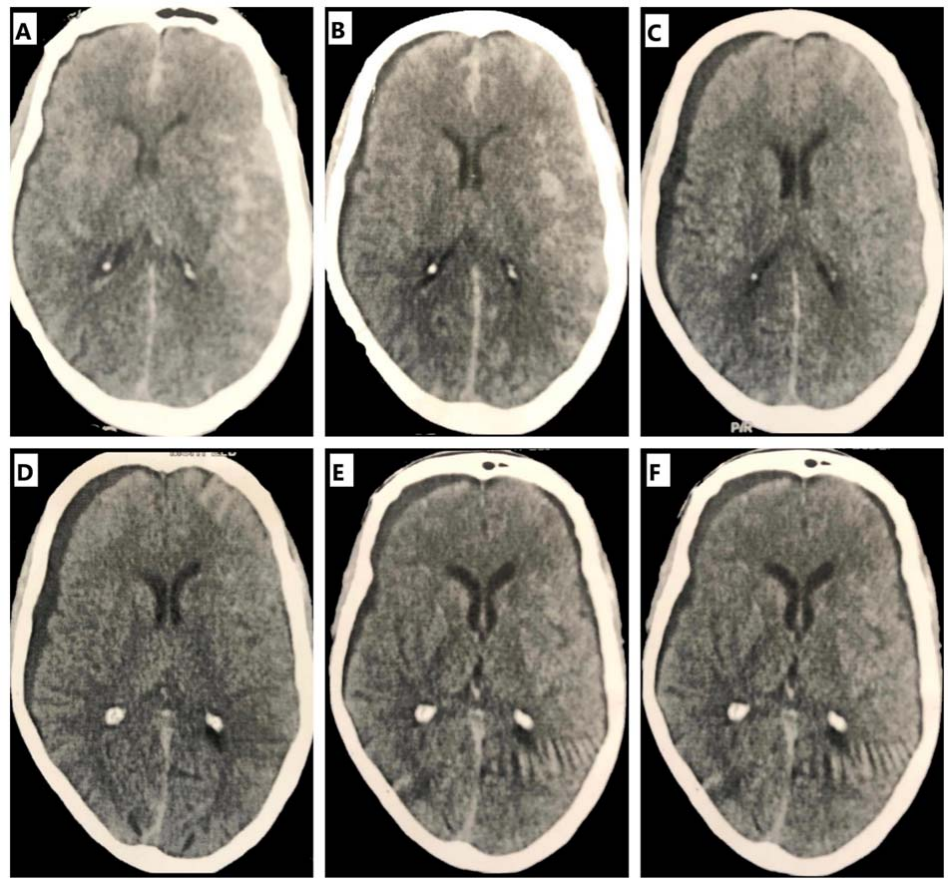
Serial unenhanced axial CT scans of the brain. (**A**) First CT after coiling at day 2. (**B**) At day 3, showing right subdural hygroma on the opposite side of leaking aneurysm. (**C**) At day 5, showing increased hygroma and no signs of ventriculomegaly. (**D**) At day 10, showing more increased hygroma and mild mass effect. (**E**) At day 14, showing newly developed left small hygroma. (**F**) At day 30, showing the regressing right hygroma.

SH can complicate the course of subarachnoid hemorrhage (SAH), particularly if there is a history of head trauma, CSF diversion techniques, or craniotomy and clipping of aneurysms. However, SH is rarely reported after endovascular coiling for ruptured aneurysms.

## CASE REPORT

The patient was a 60-year-old male with a history of hypertension and tobacco smoking. He was admitted to the intensive care unit (ICU) with acute onset of headache and mild right-sided weakness. The patient had neither history of head trauma nor fever before the presentation. Nonenhanced computed tomography (CT) of brain revealed extensive SAH. CT cerebral angiography revealed a ruptured left posterior communicating artery aneurysm (PCA), 4.5 × 6 mm with neck 2–3 mm, and another internal carotid artery, para-ophthalmic, unruptured aneurysm on the right side. Hunt and Hess’s scale was 3, and Glasgow Coma Scale (GCS) was 14/15. The patient developed generalized tonic–clonic convulsion that was controlled by midazolam and kept on oral levetiracetam.

Endovascular coiling was performed 20 h after the onset of symptoms, and it was an uncomplicated technique. ICU management followed the standard protocol of SAH [2].

On the 5th day, GCS decreased to 11/15 and brain CT revealed the same degree of SAH and increased SH, but without any mass effect nor hydrocephalic changes. Transcranial Doppler to exclude cerebral vasospasm was requested but not done at that time due to technical problems.

No improvement was observed in the consciousness level, which deteriorated further. On the 10th day, GCS was 9/15 with worsened right-sided weakness (grade 2). Brain magnetic resonance imaging revealed right-sided sizable SH exerting mild mass effect and left basal ganglia ischemic focus. Multidisciplinary meeting (MDM) concluded that the patient had delayed cerebral ischemia (DCI) and significant SH with mild mass effect and recommended no intervention for SH.

At the end of the 2nd week, the consciousness level improved to GCS 11/15 and weakness was grade 3. Surprisingly, imaging revealed progressive right SH and new SH on the left side, but with no mass effect from either hygromas. Another MDM recommended continuing conservative management.

By the end of 1st month, the consciousness level was regained and right-sided weakness improved to grade 4 with mild dysarthria. Brain imaging revealed that SH had decreased. The patient was discharged to a rehabilitation center (RC) after 1 month of ICU stay.

Imaging after 2 months revealed that SAH was almost cleared, both SHs were still decreasing and a small left basal ganglia infarction. The patient was discharged home from RC without any residual weakness, fully conscious but with mild dysarthria.

## DISCUSSION

SAH is a serious medical condition with an increased fatality, an incidence of 9 per 100 000 and a fatality rate within 6 months of about 60%. In aneurysmal SAH, the three most important variables related to prognosis are the early neurological state, age and the degree of extravasation on imaging. Consciousness is the most important determinant for outcome after SAH [3].

In 1932, Dandy described SH as a collection of nonblood fluid in the subdural space that can be encapsulated under a thin membrane [4]. The pathogenesis of SH is still unclear; however, two postulated mechanisms include a tear in the arachnoid membrane causing CSF influx or decreased intracranial pressure leading to passive effusion [5, 6].

Both external hydrocephalus and SH have unique clinical entities. External hydrocephalus is usually associated with disturbances in CSF flow caused by the imbalance between production and absorption [7]. Radiographically, external hydrocephalus and SHs are similar, but ventriculomegaly is the primary distinguishing criterion [1].

Our case was managed by simple endovascular coiling through the femoral approach with the intervention of bleeding left PCA aneurysm (four coils were deployed), and the right one was postponed after complete patient recovery.

The serial imaging of our case never showed any ventriculomegaly during the course. This made it a unique case of SH post-coiling. A previous study reported a case of external hydrocephalus as it was associated with ventriculomegaly, which also complicated the course of coiling for ruptured aneurysm. However, the case remained asymptomatic and the collection resolved spontaneously [8].

Subdural collections may be seen after clipping of ruptured aneurysmal SAH but rarely seen after coiling. Ventriculoperitoneal shunting alone is an effective and accepted procedure in post-clipping states. However, patients with subdural effusion that is not accompanied by hydrocephalus do not require subdural peritoneal shunt. When selecting the treatment for subdural effusion, it is important to consider the presence of hydrocephalus [9].

The management strategies for cases of SH following aneurysmal SAH patients are still undefined. Close neurological observation is indicated to follow the progression of any mass effect or conversion into a subdural hematoma.

The management of our case regarding SH was challenging. The deteriorating consciousness level put more ambiguity on the clinical impact of SH due to the incidence of DCI at the same time of increasing size of SH and its mass effect. However, the absence of ventriculomegaly with a meticulous neurological assessment directed us to continue the conservative plan.

The etiology of the SH in the presented case is unknown as there was no history of head trauma at presentation, CSF diversion technique, nor craniotomy, which represent the potential causes for SH after SAH. Whether it is related to CSF flow dynamics post-occurrence of SAH or from a direct effect of coiling (although SH was mainly on the opposite side of bleeding aneurysm) is still to be defined.

## CONCLUSIONS

SH may arise and become symptomatic as an unusual sequela of post-coiling of a ruptured intracranial aneurysm, in which the SH can complicate the clinical course of SAH. However, the symptomatic SH may resolve spontaneously and completely without any intervention, but needs meticulous neurological assessment and follow-up.

## References

[1] Huh P-W, Yoo D-S, Cho K-S, Park C-K, Kang S-G, Park Y-S, et al. Diagnostic method for differentiating external hydrocephalus from simple subdural hygroma. J Neurosurg 2006;105:65–70. 10.3171/jns.2006.105.1.65 16874890

[2] Connolly ES Jr, Rabinstein AA, Carhuapoma JR, Derdeyn CP, Dion J, Higashida RT, et al. Guidelines for the management of aneurysmal subarachnoid hemorrhage: a guideline for healthcare professionals from the American Heart Association/American Stroke Association. Stroke 2012;43:1711–37. 22556195 10.1161/STR.0b013e3182587839

[3] Steiner T, Juvela S, Unterberg A, Jung C, Forsting M, Rinkel G, et al. European Stroke Organization guidelines for the management of intracranial aneurysms and subarachnoid haemorrhage. Cerebrovasc Dis 2013;35:93–112. 23406828 10.1159/000346087

[4] Caldarelli M, Di Rocco C, Romani R. Surgical treatment of chronic subdural hygromas in infants and children. Acta Neurochir 2002;144:581–8. 12111491 10.1007/s00701-002-0947-0

[5] Liu YG, Xu CJ, Zhu SG, Jiang YQ, Li G, Li XG, et al. Traumatic subdural hygroma developing into chronic subdural hematoma. Chinese J Traumatol 2004;7:188–90. 15294120

[6] Naffziger HC. Subdural fluid accumulations following head injury. JAMA 1924;82:1751–2.

[7] Schubert R, Cardoso ER. External hydrocephalus in adults: report of three cases. J Neurosurg 1996;85:1143–7. 8929508 10.3171/jns.1996.85.6.1143

[8] Alotaibi NM, Witiw CD, Germans MR, Macdonald RL. Spontaneous subdural fluid collection following aneurysmal subarachnoid hemorrhage: subdural hygroma or external hydrocephalus? Neurocrit Care 2014;21:312–5. 25030709 10.1007/s12028-014-0017-5

[9] Kawaguchi T, Fujita S, Hosoda K, Shibata Y, Komatsu H, Tamaki N. Treatment of subdural effusion with hydrocephalus after ruptured intracranial aneurysm clipping. Neurosurgery 1998;43:1033–8. 9802846 10.1097/00006123-199811000-00017

